# The subthalamic nucleus keeps you high on emotion: behavioral consequences of its inactivation

**DOI:** 10.3389/fnbeh.2014.00414

**Published:** 2014-12-05

**Authors:** Yann Pelloux, Julie Meffre, Elodie Giorla, Christelle Baunez

**Affiliations:** Institut de Neurosciences de la Timone, UMR7289, CNRS and Aix-Marseille UniversitéMarseille, France

**Keywords:** basal ganglia, emotion, taste reactivity, conditioned fear, ultrasonic vocalization, conditioned place avoidance, novelty-induced place preference, glucose vs. saccharine choice

## Abstract

The subthalamic nucleus (STN) belongs to the basal ganglia and is the current target for the surgical treatment of neurological and psychiatric disorders such as Parkinson’s Disease (PD) and obsessive compulsive disorders (OCD), but also a proposed site for the treatment of addiction. It is therefore very important to understand its functions in order to anticipate and prevent possible side-effects in the patients. Although the involvement of the STN is well documented in motor, cognitive and motivational processes, less is known regarding emotional processes. Here we have investigated the direct consequences of STN inactivation by excitotoxic lesions on emotional processing and reinforcement in the rat. We have used various behavioral procedures to assess affect for neutral, positive and negative reinforcers in STN lesioned rats. STN lesions reduced affective responses for positive (sweet solutions) and negative (electric foot shock, Lithium Chloride-induced sickness) reinforcers while they had no effect on responses for a more neutral reinforcer (novelty induced place preference (NIPP)). Furthermore, when given the choice between saccharine, a sweet but non caloric solution, and glucose, a more bland but caloric solution, in contrast to sham animals that preferred saccharine, STN lesioned animals preferred glucose over saccharine. Taken altogether these results reveal that STN plays a critical role in emotional processing. These results, in line with some clinical observations in PD patients subjected to STN surgery, suggest possible emotional side-effects of treatments targeting the STN. They also suggest that the increased motivation for sucrose previously reported cannot be due to increased pleasure, but could be responsible for the decreased motivation for cocaine reported after STN inactivation.

## Introduction

The subthalamic nucleus (STN) belongs to the basal ganglia. Its hyperactivity in Parkinson’s Disease (PD) leads to targeting it as a surgical treatment using High Frequency Stimulation (HFS) in PD patients (for review Benabid et al., 2006). However, STN is not only involved in motor functions (for review see Baunez and Lardeux, 2011) and various non-motor side effects have been reported in PD patients subjected to STN HFS. Among these, emotional disturbances have been reported such as decreases in physiological arousal and in the recognition of emotional expressions and subjective feelings (Péron et al., 2013). Whether or not these effects are directly due to the STN manipulation or due to modifications in the dopaminergic supply to these patients remains a matter a debate.

The STN manipulation does not necessarily imply that STN itself should be involved in emotional processes, since HFS does not only reduce electrophysiological activity of the targeted structure, but also induces orthodromic and antidromic stimulation (McCracken and Grace, 2007). This point is particularly crucial as the STN connects with structures involved in emotional processing such as ventral pallidum (VP; Haber et al., 1985), amygdala (Lambert et al., 2012) and insular cortex (Berendse and Groenewegen, 1991). Therefore, emotional disturbances seen in some PD patients under STN HFS could rise from the indirect stimulation of these latter structures. For all these reasons, it is important to better understand the contribution of STN itself in emotional processing in an intact brain, especially since STN HFS is currently applied for psychiatric disorders such as OCD (Mallet et al., 2008), and proposed for treatment of addiction (Pelloux and Baunez, 2013).

In order to clarify the specific role of STN in emotions, we investigated the effect of axonal sparing lesions of this structure (to avoid the possible stimulating effect of HFS) on relevant behavioral assets. We used the taste reactivity test, the conditioned fear and place conditioning procedures, which are sensitive to the inactivation of the structures connected with STN and involved in emotions (i.e., VP, amygdala and insular cortex) (LeDoux et al., 1990; Cromwell and Berridge, 1993; Contreras et al., 2007; Li et al., 2013). Additionally, with the purpose of controlling for the specific involvement of the STN in responses elicited by affective stimuli, we also assessed the effect of STN lesions on responding for novelty as a more neutral affective stimulus.

Simple emotions such as like and dislike are initially unconditioned responses to external or internal (homeostatic/interoceptive) stimuli relevant for the survival. These responses are cognitive (valuation) and physiological (arousal) and can be conditioned to neutral stimuli. Now, whether emotions are critical for adapted behavior remains a matter of debate. Descartes argued their overwhelming experience “can entirely eradicate or pervert the use of reason” (Descartes, 1649). In contrast, Damasio has stressed “the crucial role of feeling in navigating the endless stream of life’s personal decisions” (Damasio, 1994).

Therefore we further investigated here the behavioral consequences of possible alterations in emotional processes after STN manipulation. Emotions and homeostasis restoration constitute the driving forces behind motivation. For instance, motivation for food can be sustained by anticipation of the elicited pleasure. Considering the links between emotion and motivation, blunted emotional responding observed in PD patients with HFS should be tied with decreased motivation, although it does not seem to be necessarily the case. In fact, data obtained in rats with intact dopamine transmission have shown that STN lesion (Baunez et al., 2005) or HFS (Rouaud et al., 2010) increases motivation for sweet food. But foraging for food can also be driven by the prospect of caloric intake to restore the homeostatic balance. We therefore aimed to dissect these two features to clarify more precisely the role of STN for each aspect. For this purpose, rats were given the choice between sweet and pleasant but non-caloric saccharine solution vs. more bland but caloric glucose solution.

## Materials and methods

### Subjects

64 male outbred Long Evans rats (Janvier, Le Genest-St-Isle, France), weighing ~320 g upon arrival were housed in pairs in polycarbonate cages (*L* = 40, *W* = 25, *H* = 18 cm) and maintained under a reversed 12-h light/dark cycle (lights on at 7:00 P.M.) at a constant temperature (21 ± 1°C), with free access to laboratory chow (Scientific Animal Food and Engineering, Augy, France) and water. All procedures were conducted in accordance with the European Community Council Directive of November 24, 1986 (86/609/EEC), and the national French Agriculture and Forestry Ministry (decree 87–849).

### Surgery

Once rats reached 380 g, they were anesthetized with 30 mg (100 mg/mL, i.m.) ketamine (Imalgene, Merial, Lyon, France) and 8.5 μg (85 μg/mL, i.m.) Medetomidine (Domitor, Janssen, Issy-les-Moulineaux, France) and placed in the stereotaxic frame (David Kopf Instruments, Tujunga, US). Bilateral 30-gauge stainless-steel injector needles connected by Tygon tubing (Saint Gobain performance plastics) with a 10 μL Hamilton microsyringe (Bonaduz, Switzerland) fixed on a micropump (CMA, Kista, Sweden) were positioned into the STN. Coordinates for the aimed site were (in mm, with tooth bar set at −3.3 mm): anteroposterior −3.7; lateral ± 2.4 from bregma; dorsoventral −8.35 from skull (Paxinos and Watson, 2005). Rats were subjected to either bilateral axon-sparing excitotoxic lesions with 0.5 μL of 53 mM ibotenic acid (9.4 μg/μL, AbCam Biochemical, Cambridge, UK; STN-lesioned group) or received bilateral injections of the vehicle solution (phosphate buffer, 0.1 M; sham control group) over 3 min. At the end of surgery, Medetomidine was reversed by 0.2 mg (4.28 mg/mL, i.m.) of atipamezole (Antisedan, Janssen).

## Experiment 1: reactivity to neutral affective stimulus: novelty induced place preference

### Apparatus

A gray Perspex enclosure (70 × 30 × 40 cm) consisted of two main compartments (30 × 30 cm), which could be isolated by closing a sliding door located in a central compartment (10 × 10 cm). One main compartment had gray smooth floor, and its walls with vertical black and white stripes. The other had a gray non-slip treads plate and the front wall covered with black spots over a white background. A camera (PNP-90 TL, Swann Communications Ltd, Central Milton Keynes, UK) connected to a DVD recorder was mounted above the apparatus.

### Procedure

Half of the rats in the STN lesioned (*n* = 14) and in the sham control (*n* = 10) groups were habituated to one compartment and the other half to the other compartment over two 30 min sessions, performed 24 h apart. On the third day, animals were placed in their familiar environment for 30 s and the door was opened, giving rats free access to the new compartment over a 15 min session. The times spent in each compartment were measured in sec visually from video recording. The time spent and the number of entries into the three compartments were compared between Sham and STN lesioned animals using two-way mixed ANOVA with group as between subject and compartment as within subject variables (repeated measures).

## Experiment 2: affective responses to hedonic stimuli: taste reactivity to sucrose

### Surgery

The same animals were then anesthetized as described above and implanted with an intra-oral fistula according to McCutcheon et al. (2012).

### Apparatus

A transparent cylinder (ID: 19 × H: 30 cm high) was elevated 0.5 cm from a Plexiglas floor under which a camera was mounted and connected to a DVD recorder. A 10 mL syringe mounted on a pump (Razel, St. Alban’s, USA) could be connected to the fistula via tygon tubing.

### Procedure

Once connected, rats were placed into the cylinder for 2 min. The pump was then activated for 30 s delivering 1 ml passive infusion of water for habituation. Habituation was repeated the following day. On day 3, three-30 s infusions of increasing concentrations of sucrose (4, 10 and 32% w/v) were then performed, interspaced with 2 min wash out periods. Hedonic (rhythmic tongue protrusions) and aversive responses (nose rubbing, wet dog shakes and gapes) were later counted manually over the 30 s infusions from video recording. Scores were square root transformed, as the number of hedonic responses elicited by intra-oral sucrose infusions did not comply normal distribution and for consistency with scoring of aversive responses. The impact of the lesion was evaluated with a two-way mixed ANOVA with the concentration as the within-subject factor (repeated measures) and groups as the between-subject factor.

## Experiment 3: calories vs. pleasure of sweetness: operant choice between glucose and saccharine

### Apparatus

Four operant conditioning chambers (29.5 × 32.5 × 23.5 cm; Med Associates, Georgia, VT, USA) were each placed within a sound and light-attenuating cubicle and controlled by MED IV PC software. Two retractable levers were mounted in one sidewall 12 cm apart and 8 cm above the grid floor. A house light was located on the ceiling. On the opposite wall, two recessed magazines (3.8 × 3.8 cm wide, 5.5 cm from the grid floor), fitted with infrared photo-cells, were each connected *via* Tygon tubing to a 10 mL syringe mounted on a pump.

### Procedure

Twenty rats (12 lesioned, 8 sham-operated) from a different cohort were kept food restricted during all the procedure (14 g/day) and were initially water restricted (free access water for 1 h per day). Rats were first trained to nose-poke into the recessed magazines for water (0.1 mL) over two 20 min-sessions. Subsequently, animals were required to press levers for water over 20 min-sessions. Then, choice procedure between saccharine and glucose solutions began with 4 sampling trials inter-spaced by 1 min time-outs. For each sampling trial, only one of the two levers was available and once pressed, it was retracted and the corresponding reward delivered (either 0.1 ml of saccharine (0.2% w/v, Sigma-Aldrich, Steinheim, Germany) or glucose (Sigma-Aldrich), counterbalanced among rats. On the following sampling trial, the other lever was presented and this sequence of 2 sampling trials was repeated once. Then, for 20 trials, both levers were inserted. Responding on one lever resulted in the retraction of both levers and the reward chosen delivered in the corresponding receptacle. The concentration of the initial 4% glucose solution was increased by 2% every 10 days (to 6 and 8% w/v). Then, in order to favor learning of the caloric value of the solutions, the animals were totally food deprived and given free access to 8% glucose solution overnight (from 18:00 to 9:00) in their home cage and similarly to saccharine solution the following night (Forced consumption procedure). Afterwards, rats were returned to their normal restricted diet and to the choice procedure for 5 consecutive daily sessions. The sequence of <2 overnight incentive conditioning periods followed by 5 choice sessions > was done 5 times in total. Five last sessions during which choices were unlimited and available for 40 min were performed to test satiation effect.

For all phases, choices were expressed as saccharine preference ratio calculated by the average number of choices for saccharine *minus* choices for glucose on the total number of trials, averaged across 5 days. The effect of increasing the glucose concentration on saccharine preference in STN lesioned and Sham animals was assessed across 2 blocks of 5 consecutive days per concentration with a three-way mixed ANOVA with 5 day-blocks and concentration as the within-subject factors (repeated measures) and group as the between-subject factor. The effect of incentive conditioning sessions on saccharine preference in STN lesioned and Sham animals was analyzed across the blocks 6 to 11 with a two-way mixed ANOVA with 5 day-blocks as the within-subject factor (repeated measures) and group as the between-subject factor. Then the effect of extending choice sessions on saccharine preference in STN lesioned and Sham animals was analyzed across the blocks 11 to 12 with a two-way mixed ANOVA with 5 day-blocks as the within-subject factor (repeated measure) and group as the between-subject factor. The effect of STN lesion on glucose or saccharine consumption during the forced consumption procedure (incentive conditioning) was tested statistically using a three-way mixed ANOVA with substance and sessions as within subject factors (repeated measures) and groups as between subject factor.

## Experiment 4: affective state elicited by interoceptive aversive stimulus: conditioned place aversion induced by lithium chloride

### Apparatus

were similar to those used in experiment 1 with all walls gray and spatial organization of gray Perspex columns differing between compartments (Rouaud et al., 2010).

### Procedure

One week after the choice experiment and ad libitum regimen, rats could first explore freely both compartments for 15 min for 2 days. The time spent in each compartment on the second day was measured. The following day, all rats received lithium chloride (LiCl) (63 mg/kg; 10 ml/kg, i.p., Sigma-Aldrich), were left in their home cage for 5 min before experiencing one of the compartment for 30 min. The next day, the same sequence was applied with saline solution (NaCl 0.9%) and the other compartment. LiCl and saline conditioning were repeated for a total of three sessions for each. The compartment associated with the LiCl was counterbalanced across subjects.

After conditioning, the animals were again given free access to both compartments for 15 min. The time spent in each compartment was measured. The score of preference/avoidance was calculated as the difference between the time spent in the LiCl-paired compartment on the testing day and the time spent in the same compartment on the first day, before conditioning. Evidence for avoidance was effective when time spent before and after conditioning were significantly different using a paired *T* test. These scores were further compared between Sham and Lesioned animals using a Student’s *T* test.

Video-recording of conditioning and test sessions were later analyzed to assess the horizontal and vertical (number of rearings) activity after LiCl or saline injections. For the measurement of the horizontal locomotor activity, two lines separated the compartment in four equal squares and the number of times the base of the tail crossed the lines was manually counted.

## Experiment 5: affective state induced by exteroceptive aversive stimulus: fear conditioning induced by electric foot shock

### Apparatus

Two operant conditioning chambers, as previously described, had floors consisting of bars, spaced at 1 cm, which were connected to a scrambler shock generator (Med associates, Georgia, VT).

A camera and a frequency division ultrasound detector (bat baton®, Batbox, Steyning, UK) (allowing the incoming frequencies to be divided by ten, thereby bringing USV within the human hearing range) were mounted on the ceiling and connected to a DVD recorder for later scoring of freezing behavior and 22 kHz ultrasonic vocalizations (USV) as two indices of negative affective states. We focused indeed on low frequency (22-kHz) USV as they indicate a negative affective state, since they are emitted during various unconditioned and conditioned aversive situations and their playback can induce avoidance (for review see Schwarting and Wöhr, 2012).

### Procedure

On day 1, 3 and 5, each of the 20 rats of a third cohort (12 lesioned and 8 shams) was placed in a conditioning box. The houselight was turned on and two minutes later, a single foot shock (2 s, 0.5 mA) was delivered. After an extra 2 min period the houselight was turned off and the animal was returned to its home cage. During the days following conditioning (that is day 2, 4 and 6), the animal was returned to the chamber for a 4 min test session during which only the houselight was lit. Shock reactivity, USV and freezing behavior during conditioning or post-conditioning sessions were later manually quantified from recordings. For assessment of shock reactivity, 2 lines divided the chamber in four equal squares and the number of times the basis of the tail crossed the lines during shock was manually counted. The percent time freezing was calculated based on the number of times rats were found completely immobile at 5-s spaced monitoring on the total number of observations.

All measures were analyzed using a 2-way mixed analysis of variance (ANOVA) with group (Sham control and STN lesioned) as between subject factor and sessions as within subject factor (repeated measures). For qualitative analysis of 22 kHz USVs the duration of all post shock 22 kHz USVs were determined using Avisoft SAS Lab Pro software and their distribution compared between Sham and STN lesioned animals using Khi^2^. The paucity of ultrasonic vocalizations over test sessions (day 2, 4 and 6) could not allow similar analysis.

### Histological assessment

At the end of the experiments, rats were euthanized with pentobarbital sodium (Dolethal, Vetoquinol, Lure, France, 73 mg/mL, 1.5 mL i.p.). Their brains were removed, frozen in isopentane (Sigma-Aldrich) and kept at −80°C; they were then sectioned coronally at 50 μm thickness in a cryostat. Sections were later stained with Cresyl Violet (Fluka-Chemika).

## Results

### Histology

**Figure 1:**
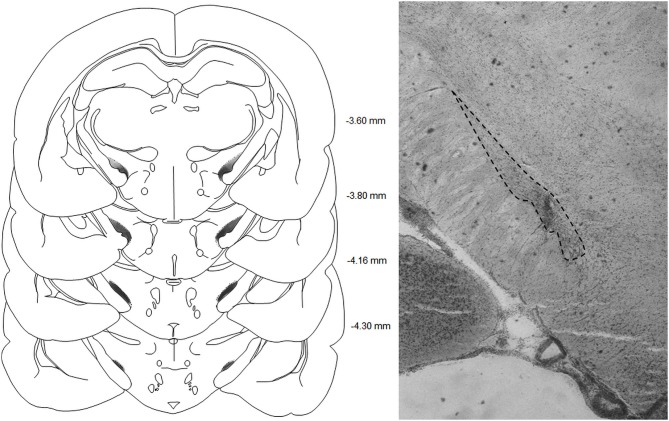
**Left panel:** schematic representations of the subthalamic lesions on standardized sections of the rat brain. The numbers adjoining each section refer to distances from bregma (adapted from Paxinos and Watson, 2005).

The sites and extent of lesions were characterized by a neuronal loss and associated gliosis and are illustrated in Figure 1. In total, 9 out of the 38 rats were excluded from the STN lesioned group for unsatisfactory lesions, being either too restricted or outside the STN.

### Experiment 1: reactivity to neutral affective stimulus: novelty induced place preference (NIPP)

**Figure 2:**
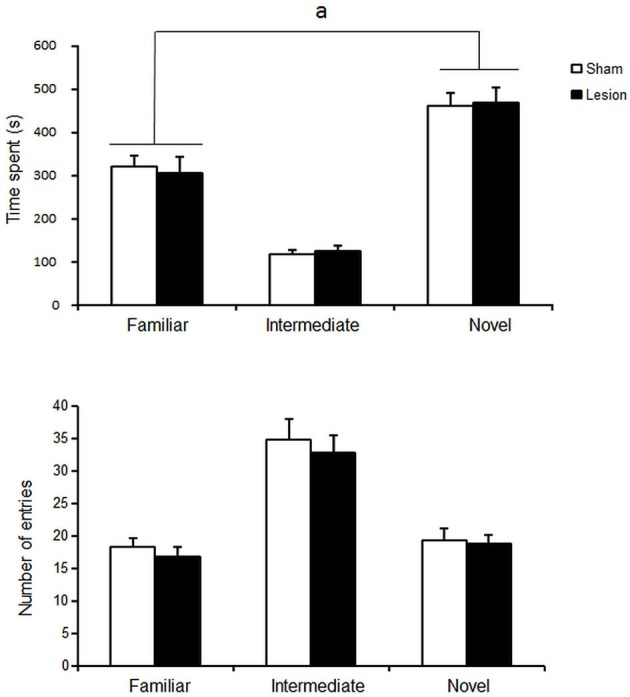
**Mean ± SEM of the time spent (upper panel) and total number of entries (lower panel) in the different compartments over 15 min of sham Control (white histograms s, *n* = 10) or STN lesioned animals (black dots, *n* = 13).** “a”: Fisher’s LSD *p* < 0.05.

STN lesioned and Sham control groups spent more time in the novel compartment than in any of the other compartments [differences between compartment *F*_(2,36)_ = 54; *p* < 0.001] but at similar levels [no interaction < compartment > *x* < group > *F*_(2,36)_ < 1; NS] (Figure 2, upper panel). No difference was observed between groups in the number of entries into the different compartments [group effect *F*_(1,36)_ < 1, NS] (Figure 2, bottom panel).

### Experiment 2: affective responses to hedonic stimuli: taste reactivity to sucrose

**Figure 3:**
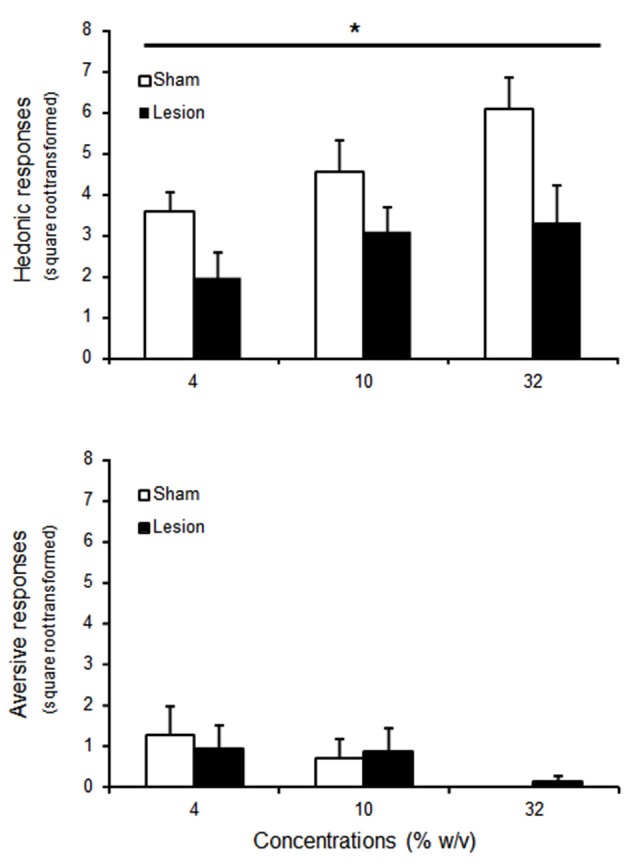
**Mean ± SEM of square root transformed number of hedonic (tongue protrusion, upper panel) and aversive (nose rubbing, wet dog shakes and gapes, bottom panel) responses to intra-oral passive infusion of increasing concentrations of sucrose for 9 animals per group (sham: white histograms; lesioned rats : black histograms)**. “*” Repeated ANOVA Main effect of group *p* < 0.05.

During the taste reactivity test, one sham and two lesioned rats were excluded due to failure of the intra-oral fistula. For the remaining animals, increasing the concentration of sucrose increased the number of tongue protrusions (Figure 3A), while decreasing the relative low number of aversive responses to the infusion of the substance (Figure 3B) [Concentration effect *F*_(2,30)_ = 7.2; *p* < 0.01] and *F*_(2,30)_ = 6.9; *p* < 0.05 respectively]. STN lesioned rats showed however blunted hedonic responses whatever the concentration used when compared to sham animals [Group effect *F*_(1,30)_ = 5.7; *p* < 0.05], while presenting unaffected aversive responses [Group effect *F*_(1,30)_ < 1; NS].

### Experiment 3: calories vs. pleasure of sweetness: operant choice between glucose and saccharine

**Figure 4:**
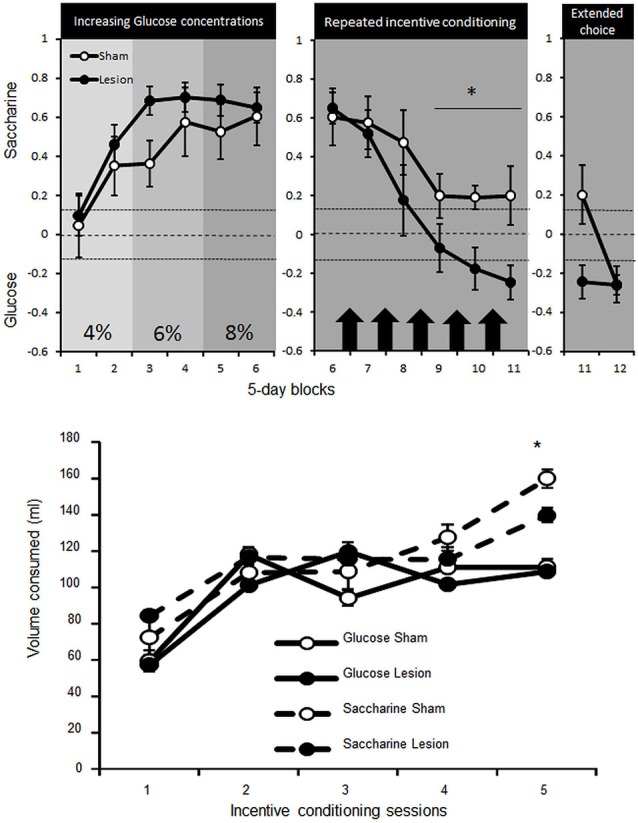
**Upper panel:** mean ± SEM of the preference for saccharine (positive score) or glucose (negative score) solutions (expressed as the number of choice for saccharine—choices for glucose on the total number of choices) for Sham control (white dots, *n* = 5) or STN lesioned rats (black dots, *n* = 10) over 5-day-blocks. In the left panel saccharine preference is represented over the course of increasing concentration of glucose (2 blocks of each concentration: 4, 6 and 8%). The central, panel represents the preference for saccharine before (block 6) and after incentive conditioning (represented by black arrows, Blocks 7–11) where animals could learn separately the caloric value of saccharine or glucose over two consecutive nights. The right panel then illustrates the preference for saccharine during block 11 when access to glucose was limited and the 5 session block with unlimited trials for 40 min (Block 12). The dashed lines represent the indecision point and any values above the upper dotted line correspond to significant preference for saccharine while any values below the lower dotted line represent significant preference for the glucose solution. Differences between Sham and STN lesioned animals “*” Fischer’s LSD *p* < 0.05. **Bottom panel:** mean ± SEM of the amount of glucose (straight lines) or saccharine (dashed lines) consumed by Sham (white dots) or STN lesion rats (black dots) overnight (from 7:00 PM to 9:00 AM )in their home cage when only one substance was available and rats were totally food deprived. Differences between Sham and STN lesioned animals “*” Fischer’s LSD *p* < 0.05.

Two Sham animals were excluded from the experiment as they were unable to learn to lever press for water. The remaining rats progressively developed a preference for the non-caloric, but sweet, saccharine over caloric glucose solution, from the 2nd 5-day-block onward [paired Student’s *T* tests *T*s_(74)_ > 9.9; *p*s < 0.001], despite being food restricted and offered increasing concentrations of caloric glucose solution [Concentration effect *F*_(5,65)_ = 26.3, *p* < 0.01]. This preference appeared in both STN lesioned and sham control rats [no interaction Group × Concentration *F*_(2,24)_ = 1.2; NS] (Figure 4, upper left panel).

Under food deprivation and free access to glucose or saccharine (on alternate nights) in their home cage, rats increased consumption of both substances [Session effect *F*_(4,52)_ = 95,2; *p* < 0.001]. The consumption was nevertheless more pronounced for saccharine [interaction Substance × Session *F*_(4,52)_ = 6.1; *p* < 0.001] and especially for sham compared to STN lesioned rats [interaction Group × Substance × Session *F*_(4,52)_ = 2.6; *p* < 0.05] (Figure 4, bottom panel). Getting the opportunity to understand the caloric supply provided by glucose as a result of incentive conditioning, the animals in the choice procedure reduced progressively their preference for saccharine [Block effect *F*_(5,65)_ = 22 ; *p* < 0.001], yet consistently less in sham rats [interaction Block × group *F*_(5,65)_ = 2.5; *p* < 0.05], which still maintained preference for saccharine [paired Student’s *T* tests *T*s_(24)_ > 2.4; *p*s < 0.02], while STN lesioned rats switched their preference for the glucose solution [*T*s_(49)_ > 5.4; *p*s < 0.001 for Block 10 and 11] (Figure 4, upper middle panel).

Extended choice session to 40 min revealed that sham could also shift their choice towards glucose to reach the preference of STN lesioned animals [interaction Block × group *F*_(1,13)_ = 8.4; *p* = 0.01] for the glucose solution [*T*_(24)_ = 3.9 and *T*_(49)_ = 6.8; *p*s < 0.001 respectively for Sham and STN lesioned rats] (Figure 4, upper right panel).

### Experiment 4: affective state elicited by interoceptive aversive stimulus: conditioned place aversion induced by lithium chloride (LiCl)

**Figure 5:**
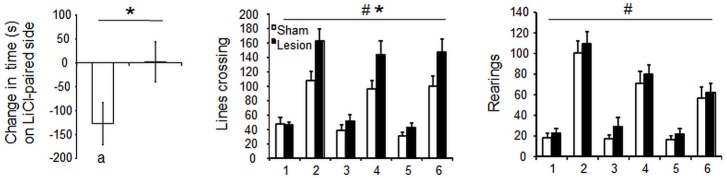
**Left panel:** mean score of preference ± SEM (i.e., the difference in the time spent in the Lithium Chloride paired compartment before and after conditioning) (left panel) in Sham (*n* = 7) and STN lesioned rats (*n* = 9). Significant difference between Sham and STN lesioned animals “*”: Student’s *T* Test, *p* = 0.05, significant place aversion : “a” paired student *t* test, *p* < 0.05. **Central and right panels:** mean horizontal and vertical locomotor activity ± SEM, over 30 min session in the compartments after the injection of Lithium Chloride (day 1,3 and 5) or saline (day 2, 4 and 6) in Sham control (white bars) and STN lesioned rats (black bars). Three way mixed ANOVA for repeated measures: “#” significant differences between Lithium Chloride and Saline, *p* < 0.0001. ”*” significant differences between sham and STN lesioned groups, *p* = 0.03.

Despite STN lesioned rats being more active than the sham controls [Group effect *F*_(1,28)_ = 5.6; *p* < 0.05], the injection of LiCl drastically decreased horizontal [Treatment effect *F*_(1,28)_ = 54.3; *p* < 0.0001] and vertical [*F*_(1,28)_ = 63.6; *p* < 0.0001] activity indifferently in STN lesioned and Sham control rats [interaction Group × Treatment *F*_(1,28)_ = 3.4 and 0 respectively, *p* > 0.05] (Figure 5, lower panels). Nevertheless, the two groups significantly differed in their ability to form a LiCl place conditioning [*T*_(14)_ = 2.1; *p* = 0.05]. Indeed, Sham animals exhibited significant conditioned place aversion (CPA) [paired student’s *T*_(6)_ = 2.9; *p* < 0.03], in contrast to STN lesioned rats that did not exhibit any CPA [*T*_(8)_ < 1; NS] (Figure 5, left panels).

### Experiment 5: affective state induced by exteroceptive aversive stimulus: fear conditioning induced by electric foot shock

**Figure 6:**
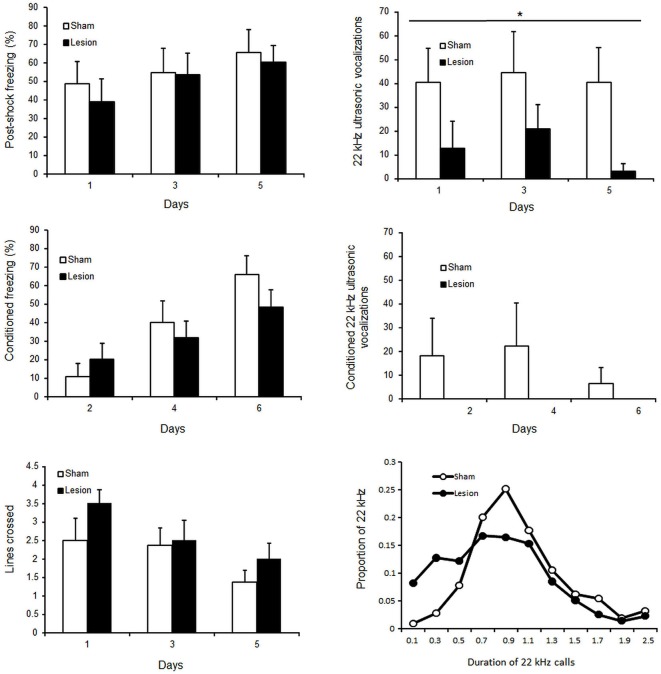
**Upper panels:** mean ± SEM of percent time freezing (left panel) and number of 22 kHz ultrasonic vocalizations (right panel) following shock (during conditioning sessions on day 1,3 and 5). **Central panels:** mean ± SEM of percent time freezing (left panel) and number of 22 kHz ultrasonic vocalizations (right panel) during simple reexposure to the aversive context (during post conditioning sessions on day 2,4 and 6). **Bottom panels:** mean ± SEM of number of lines crossed over the 2 s foot shock presentations (left panel), and proportion of the calls according to their duration (right panel). The white bars or white circles represent the scores for sham control and the black bars or black circles those for STN lesioned rats. “*” Two way mixed ANOVA for repeated measures Main effect of group *p* < 0.05.

Across conditioning sessions, rats emitted steady USV [No Session effect *F*_(2,30)_ = 0.7; *p* > 0.05], while evidencing increased time spent freezing post shock [session effect *F*_(2,30)_ = 4.9; *p* < 0.01] and reduced shock reactivity as indicated by their locomotor activity over the two second foot shock presentations [session effect *F*_(2,30)_ = 6.7; *p* < 0.01] (Figure 6, upper panels). Similarly, across post-conditioning sessions, USV remained stable [session effect *F*_(2,30)_ < 1; NS] while freezing to the context increased [session effect *F*_(2,30)_ = 26.8, *p* < 0.01] (Figure 6, middle panels). Sham control and STN lesioned rats expressed similar shock reactivity [Group effect *F*_(1,30)_ < 1; NS] and similar percent time freezing post shock [group effect *F*_(1,30)_ < 1; NS]. In contrast, lesioned animals emitted significantly less 22 kHz USV than sham animals [group effect *F*_(1,30)_ = 4.6; *p* < 0.05]. Qualitative differences were also observed between groups [Khi^2^(10) = 114; *p* < 0.001], the Sham animals expressing most of their calls around 1.1 s while STN lesioned animals emitted more shorter calls (<1 s) than Sham animals (Figure 6, right bottom panel).

## Discussion

In the present study, we have shown that STN lesions have little effect on neutral affective response as assessed in the NIPP. In contrast, pleasant responses to sweet solutions were diminished by the STN inactivation. Consequently, STN lesioned rats, in contrast to their sham counterparts, preferred blander but caloric glucose solution over more pleasant but non caloric saccharine solution, once they had learned the incentive properties of glucose. STN lesions reduced the negative vocalizations emitted after electric foot-shock and precluded the formation of LiCl-induced-CPA. Nevertheless, appraisal of the aversive component of foot-shocks and the LiCl, as assessed respectively by freezing behavior and reduced locomotor activity remained unaffected by the STN lesions. All these results are in line with a diminished affective state but spared sensitivity to homeostatic signals (interoception) following STN lesions.

Classically, animals having free-choice access to novel and familiar environments fail to display an increase in corticosterone levels (Misslin and Cigrang, 1986) and spend more time in the novel compartment. In such free choice situation, novelty can even demonstrate reinforcing properties (Besheer et al., 1999). NIPP is not guided by stress but instead, by the presumably affectively more neutral rewarding effect of novelty. This was not affected by STN lesion, suggesting that the STN is not involved in the rewarding effect of novelty. The limited effect of STN lesions on NIPP contrasted with those observed on more emotionally relevant stimuli such as sucrose.

When presented sweet solutions on the lips or in the mouth, infants, monkeys or rats express rhythmic tongue protrusions. These orofacial responses are specific for pleasant solutions given that presentation of aversive, bitter or sour compounds elicits totally different orofacial responses (gapes) and other aversive responses (head shakes or nose rubbing) (Berridge, 2000). The insensitivity of these hedonic responses to dopaminergic blockade has greatly contributed to dissociate motivation or “wanting” from affect or “liking” (Berridge, 1996). The present study highlights the involvement of the STN in “liking” evidenced by blunted hedonic responding to sucrose in STN lesioned rats. This reduced hedonic process is somehow surprising considering previous studies demonstrating that inactivating the STN by either lesions or HFS increases incentive motivation for sweet food (Baunez et al., 2002, 2005; Rouaud et al., 2010). These latter results might thus be explained by an increased “wanting” after STN lesions. Our results in fact support the dissociation between liking and wanting and suggest that STN has an opposite effect on these processes. Nevertheless, it is difficult to understand how STN lesioned rats could be more motivated for sweet food, when they are unable to appreciate its pleasurable properties. We aimed to resolve this paradox by assessing the effect of STN lesion on the choice between saccharine and glucose solutions.

All animals initially expressed a strong preference for saccharine, most likely because sweetened compounds are ecologically associated with caloric intake. Nevertheless, once lesioned rats experienced the caloric value of the glucose solution and the lack of caloric value of saccharine, they shifted their preference for glucose, overriding the original association. Eventually, but later than the STN lesioned rats, sham control animals also evidenced preference for the glucose solution when the choice session was extended to 40 min. During these extended sessions, the rats directly experienced the caloric value of choosing the glucose solution and consequently shifted their preference for this option, in line with previous studies using two bottle choice procedures over long periods (Valenstein, 1967; Valenstein et al., 1967). Altogether, these results suggest that STN lesioned rats value the calories of food, much more than the pleasure elicited by it and probably to such an extent that they are in fact more motivated for it.

The blunted affect observed after STN lesion is not limited to rewards, but can be generalized to aversive stimuli. In assessing STN involvement in negative affects induced by painful stimuli in the fear conditioning experiment, we have shown that STN lesions decreased post-shock 22 kHz USV emission, an index of negative affective states (Schwarting and Wöhr, 2012). Interestingly, this was not due to reduced perception of pain since both groups responded equally to the shocks (similar locomotor activity and similar freezing responses).

During aversive conditioning, 22 kHz ultrasonic vocalizations and freezing behavior were both simultaneously expressed, consistently with previous report (Lee et al., 2001; Jelen et al., 2003). Both groups emitted much more USV after the shocks than when only exposed to the context previously associated with shocks. In fact, vocalizations at this point were so low that the lack of significance between the two groups could be explained by a floor effect. Meanwhile, freezing remained high in both conditions, evidencing that freezing and vocalizations are differently responsive to environmental factors as previously reported (Jelen et al., 2003; Graham et al., 2009). The fact that STN lesions induce dissociation between the two behavioral responses further supports the view that they may reflect separable aspects of aversive processes. Further understanding of the variable responsible for such dissociation between freezing and ultrasonic vocalizations may refine our comprehension of the involvement of the STN in affective processes. Notably, unlike ultrasonic vocalizations, which seem to mainly constitute an unconditioned response, freezing is largely a conditioned response.

It is noteworthy that this conditioning is supported by biologically predisposed associations such as those between pain and places. These are readily acquired (Garcia et al., 1968), do not require the conscious experience of the aversive event (Weinberger et al., 1984), and could be formed without participation of affective processes. In contrast, less obvious associations such as those formed in CPA induced by nausea mobilize several behavioral resources (expectation, attention, etc.) and might in fact necessitate affective processes in contrast to more biologically relevant associations such as the association between taste and nausea (Seligman, 1971). Accordingly, we observed that sham animals expressed very mild, although consistent and significant, avoidance of the compartment previously associated with malaise despite using three pairing and a high dose of LiCl. In contrast, STN lesioned rats were unable to avoid the compartment associated with malaise. If we consider that CPA induced by nausea requires affective processes, it seems logical that by reducing these processes, STN lesions may prevent the possibility of the animals to form the association between LiCl-induced sickness and the environment.

Together with the results of the glucose/saccharine choice experiment, these latter results also evidence that the effects of STN lesion not only affect the expressive component of emotion but also the cognitive processes modulated by emotion. These experiments nevertheless only studied behavioral indices of affective processes and further researches are required to characterize the effect of STN inactivation on physiological/autonomic responses.

Emotional alterations observed after STN DBS have been proposed to results from modified complex neuronal networks. Indeed, PET studies in PD patients with DBS in the STN have shown that a deficit in facial emotional recognition is correlated with changes in the activity of the structures connected to the STN and involved in emotional processes (Le Jeune et al., 2010). It could be hypothesized that the effects of the STN lesion on emotions are indirectly mediated by changes in the activity of its related structures. However, the emotional impairments we observed after STN lesions are not completely similar to those reported after inactivation of the limbic structures that project to the STN such as the insular cortex (Berendse and Groenewegen, 1991), or VP (Haber et al., 1985) or those that receive projections from the STN, such as the amygdala (Lambert et al., 2012).

Indeed, STN lesioned rats, despite having blunted hedonic responses, are able to work for sucrose, glucose or saccharine and to adapt their preferences according to caloric (homeostatic) signals. In contrast, inactivation of the insular cortex prevents the re-evaluation of goal directed action after devaluation of the goal (Balleine and Dickinson, 2000). Moreover, disruptions of the insular cortex impair conditioning induced by malaise (Dunn and Everitt, 1988) but also the feeling of malaise, that is, they impair the sensitivity to homeostatic signals (Contreras et al., 2007). In contrast, we observed that STN lesion precluded the formation of LiCl induced CPA but spared responsiveness to LiCl, as reflected by similar reduction of locomotor activity of lesioned a Sham control animals. Then, unlike insular cortex disruption, our results suggest that STN lesions interfere with affective processes without altering the perception of homeostatic signals. Then while, physiological/interoceptive states would be represented in the insular cortex (Damasio, 1994), the multiple limbic afferences to the STN would confer this structure the integrative role of interpreting this somatic marker and turn them into emotion.

In addition, the involvement of the basolateral amygdala has been consistently demonstrated in emotional learning such as fear conditioning (LeDoux, 1996), a behavior spared by STN lesion. Now, further studies are required to determine whether STN supports conditioning as evidenced in the CPA experiment by sending affective informations to the basolateral amygdala in situations where these are necessary for learning that is when associations are not biologically prepared.

Finally, it is not surprising that STN lesions blunt responses to positive stimuli since the STN is also connected to the VP. The role of VP in these processes has been well documented. Indeed, stimulation of the VP elicits 50 kHz USV, as an index of positive emotional state (Burgdorf et al., 2007). Inversely, lesioning the VP not only abolishes pleasant responses, but it also elicits aversive responses to intra-oral infusion of sucrose (Cromwell and Berridge, 1993). Here, we demonstrated that STN lesion did not produce aversion to sucrose infusion and even decreased negative affect in different paradigms. This suggests that while VP is preferentially involved in positive affect, the STN plays a more general and complex role on affective processes, in line with the proposed role of STN as an emotional coordinator (Péron et al., 2013).

Because of its involvement in motor and non-motor processes, STN is the target for treatment of some neurological and psychiatric disorders. Therapeutic application of HFS of the STN improves motor symptoms in patients suffering from PD (Benabid et al., 2006). Preclinical studies have shown that lesioning the STN reduced similarly motor abnormalities in animal models of PD (Bergman et al., 1990; Baunez et al., 1995). These concordant effects suggest that HFS may *in fine* inactivate the STN. Furthermore, STN HFS can also treat obsessive compulsive disorders (OCD; Mallet et al., 2008). Despite these beneficial effects, STN HFS also induces behavioral side effects such as hypomania and impulsivity, as well as deficits in facial emotion recognition and modification of subjective emotional experience (for review see Péron et al., 2013). Accordingly, STN activity is affected by emotional stimuli as measured in patients suffering from PD or OCD (Buot et al., 2013). However, side effects of STN HFS could originate from orthodromic and antidromic stimulations of downstream or upstream structures of the STN. Our present results suggest however that blunted affective processes, observed in some patients treated with STN HFS, originate from the direct inactivation of the structure as evidenced here by excitotoxic lesion.

This present study therefore highlights the critical involvement of the STN in affective processing and helps explain some of the emotional side effects reported in patients under STN HFS, such as apathy encountered in PD patients (for review Rodriguez-Oroz et al., 2012; Thobois et al., 2013) but may also help to better anticipate them in some patients. On the other hand, in some situations, emotional blunting may help in overcoming affective bias, thereby promoting adaptive behavior. These latter results may account for the therapeutic potential of inactivating the STN in pathologies characterized by exacerbated affective processes such as OCD, as well as in addictive disorders where disturbed emotions such as craving and anxiety contribute to the maintenance of drug use.

## Authors contribution

Yann Pelloux and Christelle Baunez designed the experiments, Yann Pelloux, Julie Meffre and Elodie Giorla ran and analyzed the experiments and Yann Pelloux, Julie Meffre and Christelle Baunez wrote the manuscript.

## Conflict of interest statement

The authors declare that the research was conducted in the absence of any commercial or financial relationships that could be construed as a potential conflict of interest.
